# Admittance to Wildlife Rehabilitation Centres Points to Adverse Effects of Climate Change on Insectivorous Bats

**DOI:** 10.3390/biology12040543

**Published:** 2023-04-03

**Authors:** Valeria B. Salinas-Ramos, Alessandra Tomassini, Fabiana Ferrari, Rita Boga, Danilo Russo

**Affiliations:** 1 Laboratory of Animal Ecology and Evolution (AnEcoEvo), Dipartimento di Agraria, Università degli Studi di Napoli Federico II, via Università 100, 80055 Portici, Italy; 2 Tutela Pipistrelli APS, via Valeria Moriconi 52, 00138 Roma, Italy; 3 Piacenza Wildlife Rescue Centre, 29120 Niviano di Rivergano, Italy; 4 C.R.A.S. Rimini-Corpolò, via Baracchi 47, 47923 Corpolò, Italy

**Keywords:** bats, climate change, ecosystem services, rehabilitation

## Abstract

**Simple Summary:**

Every summer, in Italy and other temperate regions, many bats fall from their shelters (roosts), and, especially in urban areas, they are rescued by the public and admitted to wildlife rehabilitation centres. Why this happens is unclear, but bats might be victims of heatwaves due to climate change causing hyperthermia and dehydration. We used 5842 records provided by four Italian rehabilitation centres covering >20 years and found that for both the total sample and most species, the number of admitted bats strongly increased in the summer weeks in which temperatures rose above 30 °C. The species involved were those living in urban areas, and the effect was, in most cases, evident for both juveniles and adults. Although our study relies on correlative evidence, heat stress is the best explanation for peaks of bats falling from roosts. Bats admitted to rehabilitation centres are likely a small fraction of all those affected, so heatwaves might have important negative effects on bat populations in cities, where these mammals eat many harmful insects, providing great benefits to people. We urge that large-scale monitoring of bats in urban areas is carried out to unveil the causal mechanisms and dynamics behind this phenomenon and inform bat conservation.

**Abstract:**

Climate change is exerting a broad range of (mostly adverse) effects on biodiversity, and more are expected under future scenarios. Impacts on species that deliver key ecosystem services, such as bats, are especially concerning, so their better understanding is key to preventing or mitigating them. Due to their physiological requirements, bats are especially sensitive to environmental temperatures and water availability, and heatwave-related mortality has been reported for flying foxes and, more anecdotally, other bat species. For temperate regions, to date, no study has highlighted an association between temperature extremes and bat mortality, mostly due to the difficulty of relying on data series covering long timespans. Heatwaves may affect bats, causing thermal shock and acute dehydration so bats can fall from the roost and, in some cases, are rescued by the public and brought to wildlife rehabilitation centres (WRCs). In our work, we considered a dataset spanning over 20 years of bat admittance to Italian WRCs, covering 5842 bats, and hypothesised that in summer, the number of admitted bats will increase in hotter weeks and young bats will be more exposed to heat stress than adults. We confirmed our first hypothesis for both the overall sample and three out of five synurbic species for which data were available, whereas hot weeks affected both young and adults, pointing to an especially concerning effect on bat survival and reproduction. Although our study is correlative, the existence of a causative relationship between high temperatures and grounded bats is still the best explanation for the recorded patterns. We urge such a relationship to be explored via extensive monitoring of urban bat roosts to inform appropriate management of bat communities in such environments and preserve the precious ecosystem services such mammals provide, especially insectivory services.

## 1. Introduction

Extreme events caused by climate change, including wildfires, drought and heatwaves (prolonged periods of excessive heat [1]), are exerting adverse effects on the survival, reproduction and ecological functions and dynamics of wildlife populations [2,3]—a situation that is predicted to worsen in future decades [4].

Negative responses are especially likely to occur in animal species whose life cycles’ crucial phases are directly or indirectly affected by ambient temperatures. Bats are certainly among such species, yet observed, clear responses are still limited, and much evidence comes from model-based predictions based on future scenarios [5]. Some thermophilic bat species, such as *Pipistrellus kuhlii* [6] and *Hypsugo savii* [7], appear to benefit from rising temperatures by expanding their geographical distribution. However, many other sensitive bat species are or will be affected adversely by warmer temperatures’ direct or indirect consequences [5,8]. As facultative heterotherms, bats are especially selective towards specific roost temperatures for hibernation and reproduction [9,10] so climate change may decrease the availability of suitable roosting sites and reduce bat fitness. Additionally, bats do not possess effective evaporative cooling mechanisms to cope with high body temperatures [11]. Their large body surface exposes bats to significant water loss through their membranes via transpiration, so they will likely face dehydration in scenarios of increasing temperatures [12]. On a community scale, colonization of new regions by bat species that change their ranges in response to climate change may lead to interspecific competition with native bat communities [13]. 

One of the most compelling pieces of evidence that climate change is jeopardising bat survival is offered by direct mortality caused by heatwaves that expose bats to thermal shock and sudden dehydration. Overheating during unusual hot spells is therefore regarded as an important cause of mass-mortality events in bats [14]. Most examples of such die-offs come from mass mortality of flying foxes following heatwaves (e.g., [15,16]), but the problem is also serious for insectivorous bats, although little information is available. For example, a 2016 die-off involving >500 *Chaerephon plicatus* and *Taphozous theobaldi* in Cambodia was recently associated with heat stress [17]. 

In temperate regions, during the reproductive season, females of insectivorous bats select roosts that provide warmer microclimates to reduce homeothermy costs [18], advance timing of reproduction and improve juveniles’ growth [19,20]: conditions often found in buildings [21]. Consequently, warmer roosts are perceived as more attractive, yet at these sites, overheating caused by heatwaves may cause heat stress and kill many bats [22], turning such roosts into ecological traps [16].

Certain types of bat boxes, such as black ones facing south, may expose bats to overheating and kill them [22], as shown by occasionally recorded mortality events [23,24]. The heatwaves that affected Sardinia in the last decades are one of the main causes of the population crash recorded for the highly threatened Sardinian long-eared bat, *Plecotus sardus* [25]. While hot-roosting bat species such as urban bats are, in principle, more exposed to such events, these species often exhibit higher heat tolerance and evaporative cooling capacity than those roosting in cooler sites [26] and may overcome even critical heatwaves [27]. For instance, synurbic, day-roosting *H. savii* may attain skin temperatures of up to 46.5 °C [7], which allows them to exploit otherwise prohibitively hot artificial roosts (buildings). However, for these species too, pups may be less capable of coping with thermal stress and dehydration, yet collecting quantitative data on such events is very difficult. From this viewpoint, exploring the influence of environmental data such as heatwaves on bat admittance to wildlife rehabilitation centres (hereafter WRCs) provides unique insights into this otherwise difficult-to-explore issue. Bats rescued by the public and taken to WRCs have in most cases fallen from the roost (pers. obs.), after which, they are exposed to predation, vandalism, etc.; therefore, at least in the reproductive season, when temperatures are higher, many admittances might be primarily caused by heat stress grounding bats. 

This study analyses the association between heatwaves and a large sample of bats admitted to four major Italian WRCs from 2000 to 2021. We tested the following hypotheses and predictions:

1. In summer, the number of individual bats from urban colonies admitted to WRCs will be influenced by the mean maximum temperature of the week they were rescued, and higher numbers are predicted in hotter weeks.
2. Being more sensitive and less capable of moving autonomously inside a roost to select lower temperatures, young bats will be more exposed to heat stress than adults, so we predict that admittance data will be dominated by pups.

## 2. Materials and Methods

**Figure 1:**
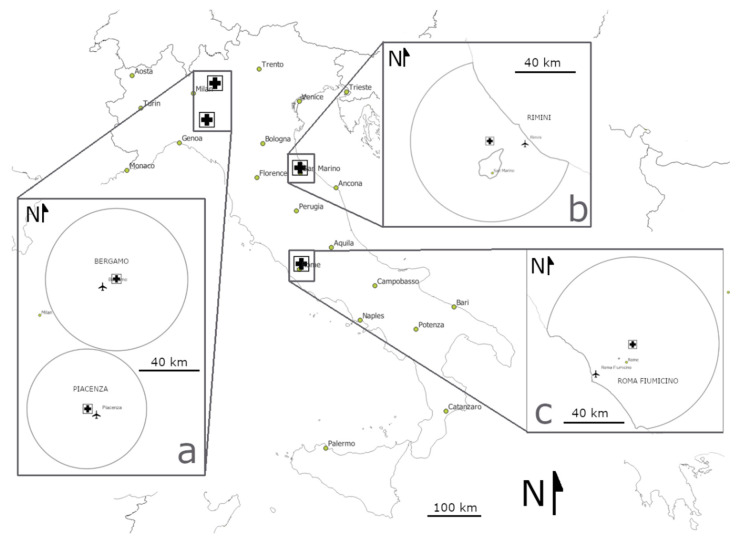
Geographical locations of the four Italian bat rehabilitation centres (indicated by crosses) and nearby airports (depicted by aeroplane icons) where temperature data were obtained. The circles indicate the primary geographic areas served by each rehabilitation centre (data provided by the respective centres). The letters identify the geographic areas where centres and airports are located. (**a**): Berga-mo-Valpredina and Piacenza; (**b**): Rimini and (**c**): Rome.

We collected data from four Italian rehabilitation centres (i.e., the Rome, Piacenza and Rimini Wildlife Rescue Centres and Natural Reserve WWF in Valpredina, Figure 1), covering 6495 bats, 4779 of which were identified to the species level (overall, 20 species). Data spanned from 2000 to 2021 (details are provided in Table S1, Supplementary Materials), but no data were available for the years 2001 and 2003. 

When admitted to centres, bats were typically distinguished as either adults or, generically, juveniles. The latter category ranged from pups to nonvolant juveniles that, although almost fully developed, could not yet forage autonomously. While recognizing newborn bats is unmistakable, almost fully developed juveniles can only be distinguished from adults based on wing bones’ epiphyseal joints, tapered in the former and knob-like in adults, and, in juveniles, shown cartilaginous sections in transilluminated wings [28]. Both species identification and age class were established by rehabilitation centre operators on admission, and only in some cases were details on stage of juvenile development provided. Likewise, individual sex was not always noted. For the scope of our analysis, we disregarded sex and classified bats as either adults or “young bats” (typically <1.5 months old). 

Causes of admittance categories differed across centres, so, for our analysis, we reclassified the data under the following categories: dehydrated/weak, fallen from roost, predation, trauma, apparently healthy, other causes and not available (NA).

We restricted this analysis to the late summer months (June through September), covering the reproductive period (birth, lactation and emancipation of juveniles). In such months, house-dwelling bats, most frequently admitted to rehabilitation centres, roost in human-made structures, where they are the most exposed to heatwaves (e.g., [25]). We used all available records for the “total” number of bats, regardless of whether a species had been identified on admittance, provided the age class was recorded. Analyses were carried out at the species level only for those species featuring >50 individuals to achieve sufficient statistical power. 

Since obtaining temperature values for the exact dates and locations of collection of all bats was not possible, as a proxy, for all bats pooled together and each single species examined, we associated the number of bats admitted to each centre per week, with the corresponding mean weekly maximum temperature recorded at the airport closest to each rehabilitation centre. In most cases, admitted bats originated from the same geographic province where the respective centre was located, so we assumed such temperature data to be a reliable proxy for the actual ambient temperatures to which the bats had been exposed before being admitted to the centres (Figure 1). 

Such mean-temperature values were then classified as either < or >30 °C, and the former category was associated with occurrence of heatwaves. This threshold reflects the general classification of heatwaves used in previous works, e.g., [25]. Temperature data were taken from www.ilmeteo.net (accessed on 14 August 2022). For weeks when no bats were collected, we still used temperature information in our analysis and entered “zero” as the number of admitted bats. 

We assessed the influences of year of admittance, centre and mean weekly maximum temperature (< or >30 °C, respectively) employing a generalized linear model (GLM) analysis of covariance (ANCOVA), where year of admittance was entered as a covariate and centre as a random factor. We used Spearman’s correlation tests to explore the relationship between the numbers of adult and young bats admitted each week. We made sure that datasets conformed to univariate test assumptions via checking their structures with a Ryan–Joiner and a Levene’s test (*p* > 0.05 in all cases). Significance was set at *p* < 0.05 and analyses were carried out with MINITAB 14 (State College, PA: Minitab, Inc.; www.minitab.com).

To support the results of the GLM ANCOVA analysis, we performed automatic linear modelling (ALM) with model selection to identify the predictors with the strongest effects on the total number of admitted bats for each species and age category (i.e., young bats or adults). We used SPSS software (IBM SPSS Statistics, Armonk, NY, USA; https://www.ibm.com/spss) with the “LINEAR” routine, applying the best subset criteria and selecting the best models based on the lowest values of corrected Akaike information criteria (AIC). We performed 15 separate ALM analyses using bat number as the response variable in the model. For each model, we divided the data based on species and bat age class and assessed the significance of experimental factors, including centre, heatwaves and years, as categorical and continuous predictors in the model.

## 3. Results

**Figure 2:**
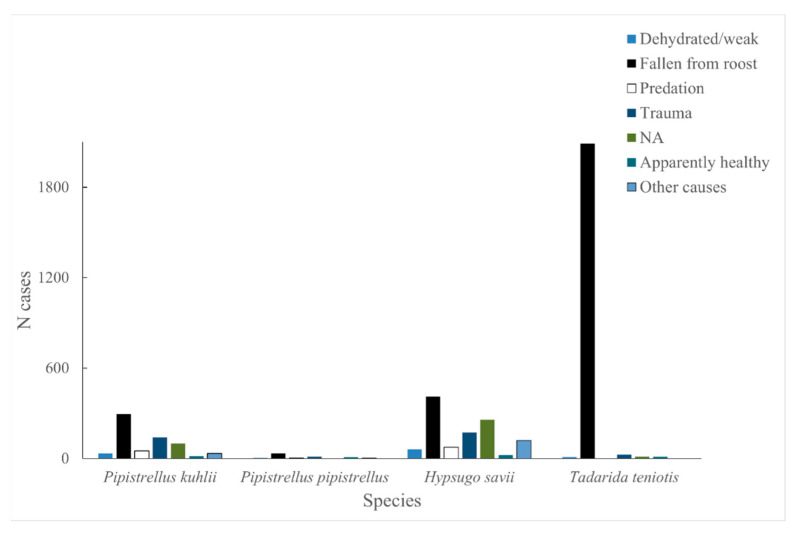
Causes of bat admittance to four Italian wildlife rehabilitation centres. In the legend, NA refers to individual bats for which no information was available.

We restricted this analysis to the 5842 bats admitted in the summers of 2000–2021 (except for 2001 and 2003, when no data were available), as well as to the only four species (*Pipistrellus kuhlii*, *P. pipistrellus*, *H. savii* and *Tadarida teniotis*) whose sample sizes were >50 individuals (Figure 1 and Table S2 in Supplementary Materials). The sample was dominated by bats recorded as “fallen from the roost”, followed by individuals showing trauma or lack of information (Figure 2). We found significant and, in most cases, strong correlations between the weekly numbers of adult vs. young bats admitted to the centres (total, r = 0.907, *p* < 0.005; *P. kuhlii*, r = 0.989, *p* < 0.001; *P. pipistrellus*, r = 0.932, *p* < 0.001; *H. savii*, r = 0.992, *p* < 0.001), with only the exception of *Tadarida teniotis* (r = 0.025, n.s.), whose sample was mostly (96%) made of young individuals.

When the total sample size was considered, the year of admittance did not influence the number of admitted bats, while the different rehabilitation centres received significantly different numbers of bats and higher numbers were admitted on weeks in which mean maximum temperatures exceeded 30 °C (Table 1). Such effects were detected both for the overall sample and for adults and young bats separately.

Based on the ALM analysis (see Table S3 in Supplementary Materials), the best models for both the overall sample and young bats included the variables “centre” and “temperature”, while for adults, all three variables—“centre”, “temperature” and “year”—were found to be important.

In hot weeks (i.e., those with a mean maximum temperature ≥ 30 °C, *n* = 197), typically, the number of admitted bats was much higher than the number recorded in cooler (<30 °C, *n* = 545) weeks: total sample, 12.5 ± 33.7 bats vs. 6.2 ± 16.1; young bats, 9.6 ± 32.9 vs. 4.5 ± 15.0; and adults, 2.9 ± 6.4 vs. 1.7 ± 3.6.

*Pipistrellus* spp. provided contrasting results. In *P. kuhlii*, the year of collection had significant effects only on the overall sample and adults, while it was not significant for young bats, whereas “centre” and the weekly mean of maximum temperatures had significant effects on all groups, with more bats admitted in hotter weeks (Table 2). 

The ALM analysis (see Table S4 in Supplementary Materials) revealed that the variables deemed significant in the GLM analysis were also included in the best models based on AIC values.

In *P. pipistrellus*, however, the number of admitted bats only differed among centres, whereas the remaining factors had no influence (Table 2), and the ALM analysis fully confirmed this outcome (see Table S5 in Supplementary Materials). In all cases, the Rome centre admitted significantly more bats than the remaining centres, according to Tukey’s post-hoc test.

In hot weeks (i.e., those with a mean maximum temperature ≥ 30 °C, n = 198), the number of admitted *P. kuhlii* was larger than the number recorded in cooler (<30 °C, n = 544) weeks: total sample, 1.9 ± 4.6 bats vs. 1.0 ± 2.9; young bats, 1.1 ± 3.7 vs. 0.5 ± 2.3; and adults, 0.8 ± 1.8 vs. 0.4 ± 1.2. For *P. pipistrellus*, however, we found no difference between hot (mean maximum temperature ≥ 30 °C, n = 198) and cooler (<30 °C, n = 545) weeks: total sample, 0.1 ± 0.4 bats vs. 0.1 ± 0.6; young bats, 0.1 ± 0.3 vs. 0.0 ± 0.04; and adults, 0.0 ± 0.1 vs. 0.0 ± 0.3.

*Hypsugo savii* provided results identical to those of *P. kuhlii*, with a significant effect of the year on adults and the overall sample and significant effects of the centre and the temperature on all categories (Table 3), and the ALM analysis fully confirmed this outcome (see Table S6 in Supplementary Materials). Again, hotter weeks corresponded to higher numbers of admitted bats, both adults and young individuals. For both species, the “centre” effect was mostly due to the highest number of bats admitted to the largest centre (Rome). For *H. savii*, too, in hot weeks (mean maximum temperature ≥ 30 °C, n = 198), more bats were admitted than in cooler (<30 °C, n = 545) weeks: total sample, 2.2 ± 4.1 bats vs. 1.2 ± 2.8; young bats, 1.2 ± 2.9 vs. 0.6 ± 2.0; and adults, 1.0 ± 2.3 vs. 0.6 ± 1.6.

Finally, *Tadarida teniotis* showed no strong effect of temperature (but both the overall sample and those of pups showed borderline significance values), while the only significant influences were those of the centre (most bats were admitted to the Rome wildlife rehabilitation centre) and the year of admittance (significant for all categories except adults, which showed a borderline *p* value) (Table 4). The borderline significance values observed for the variable “temperature” in both the overall sample and young bats were reflected in the ALM analysis (see Table S7 in Supplementary Materials). The best model for these two categories still included the variable “temperature,” along with “centre” and “year”.

## 4. Discussion

Based on a large dataset covering over 20 years, we show that, in agreement with our first hypothesis, the number of individual bats admitted to WRCs was largely affected by the mean maximum temperature of the week when they were rescued; specifically, that in hot weeks, with mean maximum temperatures over 30 °C, more bats were admitted. This pattern was clear in the overall sample, as well as for most species except *P. pipistrellus* and *T. teniotis*. The species for which we obtained a sufficiently large sample size were all synurbic, confirming that bats admitted to WRCs are in most cases rescued by the public in urban or rural areas. Although we admit that our analysis is correlative, it is difficult to conceive alternative explanations for such a strong, evident relationship, which points to a direct effect of hot temperatures on bat health. Noticeably, most admitted bats (adults and young) were recorded as having fallen from the roost, but for most other cases, too (presence of trauma, dehydration, predation), the most likely explanation is that injury occurred after the bats fell from the roost, thus being exposed to direct sun radiation, predators, etc. 

Despite the species being frequently synurbic, *P. pipistrellus* admittance records were not influenced by heatwaves, unlike *P. kuhlii* and *H. savii*. *Pipistrellus pipistrellus* is the least thermophilous of these three species and, under climate change, is predicted to shift its geographic range northwards, leaving the progressively hotter southern regions, unlike the remaining two species, predicted to expand their range northwards while persisting at lower latitudes [29]. For the same reason, *P. pipistrellus* tend to roost at higher altitudes than *P. kuhlii* in areas of sympatry and select cooler roosts, more protected from thermal extremes, such as cracks in walls, whereas the other two species actively seek hot roosts: for instance, the spaces behind sun-drenched gutters [7,30]. Ref. [30] found that in Canton Ticino (Switzerland) *P. kuhlii* roosts mainly faced south, while *P. pipistrellus* showed no preference. The less-pronounced thermophily in *P. pipistrellus* and the consequent use of relatively cooler roosts might explain why, for this species, we did not detect any admittance increase in hot weeks, whereas thermophilous bat species, being lured into thermally exposed roosts, may especially suffer from heat stress and end up on the ground. 

The case of *T. teniotis* differs from the others: these were mostly juveniles from a single summer colony in a crowded area of Rome, roosting in a narrow space between two blocks of flats on the sixth floor (Figure 1b in [21]). Such bats showed pronounced deformation and fractures of wing joints and bones, were collected by the hundreds on the ground and showed unusually high lead concentrations, perhaps one of the main factors causing their problem [31]. However, our analysis showed that at least for young bats, some influence of high temperature cannot be ruled out. 

Our second hypothesis, i.e., that young bats would be more affected than adults, was rejected: the effect of temperature influenced the admittance of both young bats and adults, suggesting that heat stress may be a serious issue decreasing colony productivity also survival. This is in line with previous occasional observations made in the Mediterranean region [32] as well as elsewhere: for instance, the >500 *C. plicatus* and *T. theobaldi* killed by a heatwave in April 2016 in a Cambodian colony comprised more adults than pups, as assessed from a subsample of 176 dead bats [17]. Overall, the picture we provide, as well as such previous observations, are consistent with significant disruption of demography in bat colonies affected by overheating. On such a basis, even the strong thermal resilience characterising hot-roost dwellers (e.g., [7]) may not suffice to cope with the strong impact of heat stress.

Both “centre” and “year of admittance” proved statistically significant in most cases; while the difference among centres was not driven by environmental factors and was due to the much larger catchment area of certain centres over others (for example, the Rome WRC received far more bats than all others), different years may have experienced different frequencies in heatwaves, but we refrained from exploring such potential patterns due to the likely dominant “social” effects. For instance, the Italian public’s awareness of bat importance has increased dramatically in the last two decades, so many more bats have been rescued and admitted to WRCs in recent years than in the past (A. Tomassini, pers. comm.). While the numbers of admitted bats and the duration of coverage varied among the rehabilitation centres, we have no reason to believe that these differences had any impact on the overall pattern we have uncovered.

Although our study could not prove the existence of a direct relationship between grounded bats and overheating, it offers the first large-scale evidence of an association between bat health and heat stress. This confirms observational and experimental evidence gathered from bat-box monitoring [22,32] and suggests that the phenomenon is more widespread than previously thought. Natural roosts are a relatively rare resource, so ever-growing urbanisation has provided bats with alternative, artificial roosts that mimic natural conditions; a wall crack resembles a rock crevice, hence, it is plausible that a transition from roosting in crevices to roosting in buildings has occurred in the long history of human–bat coexistence [33]. However, while roosting sites are more abundant in buildings than in nature, buildings offer a low diversity of roosting conditions [21] and, consequently, a narrower range of microclimates to exploit. For instance, a single colony of big brown bats (*Eptesicus fuscus*) roosting in rock crevices in Canada used 72 roosts during pregnancy, lactation and postlactation, selecting crevices with specific features based on microclimate requirements and defence from predators [34]. Clearly, this is a “luxury” urban bats cannot afford, so they are constrained to higher roost fidelity [35] and consequently more exposed to sudden microclimatic changes dictated by heatwaves. Microclimate selection is vital; in the reproductive season, warm roosts may reduce the cost of homeothermy, since females cannot use prolonged torpor without delaying embryo development and inducing reduction in milk production (e.g., [36,37,38]). Especially heat-tolerant bats may therefore exploit urban roosts exposed to high temperatures and maximise the thermal benefits of a warm roosting environment [7]. However, in the climate change era, this hard-wired tendency to select high roosting temperatures turns into a trap for urban bats and may cause significant mortality, while low roost availability and/or diversity may discourage roost switching to escape deadly overheating. 

Especially in climatic conditions such as those of the Mediterranean region, where average summer temperatures are rising year by year [32], we urge that investigations be carried out at a sufficiently large number of roost sites to better comprehend the physiological and ecological dynamics that control this phenomenon and help conceive appropriate mitigation measures. We believe that such studies may greatly benefit from the important contributions of WRCs, as highlighted in this and previous [39] works. Protecting urban bat communities, reducing conflicts and promoting the vital ecosystem services bats provide [40] is, today, more important than ever in a world that is progressively dominated by urban areas at the expense of natural spaces.

## Figures and Tables

**Table 1:** Results of a GLM ANCOVA on the total number of bats admitted at four Italian rehabilitation centres in 2000–2021. Temperature = weekly mean of maximum temperatures recorded.

	Total n Bats (*n* = 5842)				Young Bats (*n* = 4361)			Adults (*n* = 1481)			
Source	d.f.	F	P	R^2^ (Adj)	F	P	R^2^ (Adj)	F	P	R^2^ (Adj)
Year	1	2.87	0.091		8.16	0.004		31.43	0.000	
Centre	3	45.54	0.000		30.99	0.000		45.27	0.000	
Temperature	1	9.34	0.002		6.09	0.014		10.11	0.002	
Error	736									
				16.53%			12.19%			19.43%

**Table 2:** Results of a GLM ANCOVA on *Pipistrellus kuhlii* and *P. pipistrellus* admitted at four Italian rehabilitation centres, years 2008–2021. Temperature = weekly mean of maximum temperatures recorded.

***Pipistrellus kuhlii***										
	**Total n Bats (*n* =900)**				**Young Bats (*n* =513)**			**Adults (*n* =387)**		
Source	d.f.	F	P	R^2^ (Adj)	F	P	R^2^ (Adj)	F	P	R^2^ (Adj)
Year	1	6.18	0.013		0.36	0.547		23.87	0.000	
Centre	3	34.90	0.000		20.09	0.000		29.30	0.000	
Heatwaves	1	7.87	0.005		5.50	0.019		4.51	0.034	
Error	736									
				14.12%			7.94%			14.29%
***Pipistrellus pipistrellus* s.l.**										
	**Total n Bats (*n* = 60)**				**Young Bats (*n* = 34)**			**Adults (*n* = 26)**		
Source	d.f.	F	P	R^2^ (Adj)	F	P	R^2^ (Adj)	F	P	R^2^ (Adj)
Year	1	0.04	0.847		0.30	0.586		0.25	0.619	
Centre	3	12.77	0.000		6.70	0.000		9.23	0.000	
Heatwaves	1	0.68	0.411		0.02	0.877		2.02	0.155	
Error	737									
				4.31%			2.01%			3.16%

**Table 3:** Results of a GLM ANCOVA on *Hypsugo savii* admitted at four Italian rehabilitation centres in 2007–2021. Temperature = weekly mean of maximum temperatures recorded.

					*Hypsugo savii*					
	Total n Bats (*n* =1068)				Young Bats (*n* = 556)			Adults (*n* = 513)		
Source	d.f.	F	P	R^2^ (Adj)	F	P	R^2^ (Adj)	F	P	R^2^ (Adj)
Year	1	6.34	0.012		1.16	0.283		9.18	0.003	
Centre	3	35.27	0.000		15.90	0.000		30.60	0.000	
Heatwaves	1	15.00	0.000		6.60	0.010		11.96	0.001	
Error	737			15.38%			7.20%			13.45%

**Table 4:** Results of a GLM ANCOVA on *Tadarida teniotis* admitted at four Italian rehabilitation centres in 2007–2021. Temperature = weekly mean of maximum temperatures recorded.

					*Tadarida teniotis*					
	Total n Bats (*n* = 2150)				Young Bats (*n* = 2066)			Adults (*n* = 84)		
Source	d.f.	F	P	R^2^ (Adj)	F	P	R^2^ (Adj)	F	P	R^2^ (Adj)
Year	1	10.08	0.002		10.71	0.001		3.56	0.059	
Centre	3	12.70	0.000		11.80	0.000		8.18	0.000	
Heatwaves	1	2.84	0.092		3.06	0.081		1.65	0.199	
Error	737			5.85%			5.62%			3.14%

## Data Availability

Not applicable.

## Supplementary Materials

The following supporting information can be downloaded at: https://www.mdpi.com/article/10.3390/biology12040543/s1, Table S1. Availability of bat admission data used for the present study across years for four rehabilitation centres in Italy.; Table S2. Summary of the number of bats (both young and adult) admitted to four Italian wildlife rehabilitation centres included in this study. Species-specific analyses were conducted only for the first four species due to their sufficient sample sizes, while the remaining species were either uncertainly classified or had small sample sizes and were therefore only included in the overall analysis of total bat numbers.; Table S3. Results of ALM (Automated Linear Modelling) with model selection on the total number of bats admitted to four rehabilitation centres. The response variable was the total number of bats, with centre, year, and temperature (>30°C) used as categorical and continuous predictors. Model selection was based on best subset selection and corrected Akaike Information Criterion (AICC). The table reports the presence or absence of each predictor in the final model. An “X” indicates the effect was included in the model, while a “-” indicates the effect was absent. ***/**/*: importance of variable as a predictor of admittance to centre. Table S4. Results of ALM (Automated Linear Modelling) with model selection on the number of *Pipistrellus kuhlii* admitted to four rehabilitation centres. The response variable was the total number of bats, with centre, year, and temperature (>30 °C) used as categorical and continuous predictors. Model selection was based on best subset selection and corrected Akaike Information Criterion (AICC). The table reports the presence or absence of each predictor in the final model. An “X” indicates the effect was included in the model, while a “-” indicates the effect was absent. ***/**/*: importance of variable as a predictor of admittance to centre.; Table S5. Results of ALM (Automated Linear Modelling) with model selection on the number of *Pipistrellus pipistrellus* admitted to four rehabilitation centres. The response variable was the total number of bats, with centre, year, and temperature (>30 °C) used as categorical and continuous predictors. Model selection was based on best subset selection and corrected Akaike Information Criterion (AICC). The table reports the presence or absence of each predictor in the final model. An “X” indicates the effect was included in the model, while a “-” indicates the effect was absent. ***/**/*: importance of variable as a predictor of admittance to centre.; Table S6. Results of ALM (Automated Linear Modelling) with model selection on the number of *Hypsugo savii* admitted to four rehabilitation centres. The response variable was the total number of bats, with centre, year, and temperature (>30 °C) used as categorical and continuous predictors. Model selection was based on best subset selection and corrected Akaike Information Criterion (AICC). The table reports the presence or absence of each predictor in the final model. An “X” indicates the effect was included in the model, while a “-” indicates the effect was absent. ***/**/*: importance of variable as a predictor of admittance to centre.; Table S7. Results of ALM (Automated Linear Modelling) with model selection on the number of *Tadarida teniotis* admitted to four rehabilitation centres. The response variable was the total number of bats, with centre, year, and temperature (>30 °C) used as categorical and continuous predictors. Model selection was based on best subset selection and corrected Akaike Information Criterion (AICC). The table reports the presence or absence of each predictor in the final model. An “X” indicates the effect was included in the model, while a “-” indicates the effect was absent. ***/**/*: importance of variable as a predictor of admittance to centre.
