# Supplementary material for: Admittance to Wildlife Rehabilitation Centres Points to Adverse Effects of Climate Change on Insectivorous Bats
Source: Biology (Basel). 2023 Apr 3;12(4):543. doi: 10.3390/biology12040543 (PMC10136049; doi:10.3390/biology12040543)
Supplement: Supplementary file 1 [file biology-12-00543-s001.zip › biology-2229157-supplementary.pdf]

Table S1. Availability of bat admission data used for the present study across years for four rehabilitation centres in Italy.

**Rehabilitation Centre**	**Years covered**
Rome	2007-2021
Piacenza Wildlife Rescue Centre	2016-2021
C.R.A.S. Rimini-Corpolò	2009-2010, 2013-2014, 2016-2021
Centro Recupero Animali Selvatici WWF Valpredina	2000, 2002, 2004-2008, 2010-2021

Table S2. Summary of the number of bats (both young and adult) admitted to four Italian wildlife rehabilitation centres included in this study. Species-specific analyses were conducted only for the first four species due to their sufficient sample sizes, while the remaining species were either uncertainly classified or had small sample sizes and were therefore only included in the overall analysis of total bat numbers.

**Species**	**Total**	**Pups**	**Adults**
Pipistrellus kuhlii	900	513	387
Pipistrellus pipistrellus	60	34	26
Hypsugo savii	1069	556	513
Tadarida teniotis	2150	2066	84
Pipistrellus sp.	1620	1179	441
Rhinolophus sp	0	0	0
Pipistrellus nathusii	1	0	1
Miniopteris schreibersii	1	1	0
Eptesicus serotinus	11	2	9
Plecotus auritus	2	0	2
Myotis myotis	1	0	1
Plecotus sp.	8	4	4
Rhinolophus hipposideros	1	1	0
Plecotus austriacus	3	1	2
Myotis capaccinii	2	0	2
Myotis myotis/blythii	1	1	0
Nyctalus leisleri	0	0	0
Rhinolophus euryale	1	0	1
Myotis emarginatus	1	0	1
Pipistrellus pygmaeus	3	0	3
Myotis crypticus	2	0	2
Myotis sp.	3	2	1
Nyctalus lasiopterus	1	0	1
Myotis daubentonii	1	1	0

Table S3. Results of ALM (Automated Linear Modelling) with model selection on the total number of bats admitted to four rehabilitation centres. The response variable was the total number of bats, with centre, year, and temperature (>30°C) used as categorical and continuous predictors. Model selection was based on best subset selection and corrected Akaike Information Criterion (AICC). The table reports the presence or absence of each predictor in the final model. An "X" indicates the effect was included in the model, while a "-" indicates the effect was absent. ***/**/*: importance of variable as a predictor of admittance to centre.

	Total		
AICC	4482.495	4483.820	4483.975
Centre**	X	X	X
Year	X	-	X
Temperature*	X	X	X
Null	-	-	X
	Young bats		
AICC	4455.790	4457.541	4460.251
Centre**	X	X	X
Year	X	X	X
Temperature*	X	X	-
Null	-	X	-
	Adults		
AICC	2085.310	2085,835	2091,148
Centre***	X	X	X
Year**	X	X	X
Temperature*	X	X	
Null		X	

Table S4. Results of ALM (Automated Linear Modelling) with model selection on the number of *Pipistrellus kuhlii* admitted to four rehabilitation centres. The response variable was the total number of bats, with centre, year, and temperature (>30°C) used as categorical and continuous predictors. Model selection was based on best subset selection and corrected Akaike Information Criterion (AICC). The table reports the presence or absence of each predictor in the final model. An "X" indicates the effect was included in the model, while a "-" indicates the effect was absent. ***/**/*: importance of variable as a predictor of admittance to centre.

	Total		
AICC	1737,808	1739,777	1741,014
Centre***	X	X	X
Year**	X	X	
Temperature*	X	X	X
Null		X	
	Young bats		
AICC	1455,016	1456,661	1456,857
Centre**	X	X	
Year	-	X	X
Temperature*	X	X	X
Null			X
	Adults		
AICC	400,347	402,024	402,034
Centre ***	X	X	X
Year**	X	X	
Temperature*	X	X	X
Null		X	

Table S5. Results of ALM (Automated Linear Modelling) with model selection on the number of *Pipistrellus pipistrellus* admitted to four rehabilitation centres. The response variable was the total number of bats, with centre, year, and temperature (>30°C) used as categorical and continuous predictors. Model selection was based on best subset selection and corrected Akaike Information Criterion (AICC). The table reports the presence or absence of each predictor in the final model. An "X" indicates the effect was included in the model, while a "-" indicates the effect was absent. ***/**/*: importance of variable as a predictor of admittance to centre.

	Total		
AICC	-1016,68	-1016,17	-1014,69
Centre*	X	X	X
Year	-	-	X
Temperature	-	-	-
Null	-	X	-
	Young bats		
AICC	-1343,36	-1341,91	-1341,5
Centre*	X	X	X
Year	-	-	X
Temperature	-	-	-
Null	-	X	-
	Adults		
AICC	-2072,09	-2017,68	-2070,13
Centre*	X	X	X
Year	X	-	X
Temperature	-	-	-
Null	-	X	-

Table S6. Results of ALM (Automated Linear Modelling) with model selection on the number of *Hypsugo savii* admitted to four rehabilitation centres. The response variable was the total number of bats, with centre, year, and temperature (>30°C) used as categorical and continuous predictors. Model selection was based on best subset selection and corrected Akaike Information Criterion (AICC). The table reports the presence or absence of each predictor in the final model. An "X" indicates the effect was included in the model, while a "-" indicates the effect was absent. ***/**/*: importance of variable as a predictor of admittance to centre.

	Total		
AICC	1639,676	1641,264	1644,006
Centre***	X	X	X
Year**	X	X	
Temperature*	X	X	X
Null	-	X	-
	**Young bats**		
AICC	1163,281	1164,386	1164,742
Centre**	X	X	X
Year		X	X
Temperature*	X	X	X
Null	-		-
	**Adults**		
AICC	821,549	822,299	828,122
Centre ***	X	X	X
Year **	X	X	
Temperature *	X	X	X
Null	-	X	-

Table S7. Results of ALM (Automated Linear Modelling) with model selection on the number of *Tadarida teniotis* admitted to four rehabilitation centres. The response variable was the total number of bats, with centre, year, and temperature (>30°C) used as categorical and continuous predictors. Model selection was based on best subset selection and corrected Akaike Information Criterion (AICC). The table reports the presence or absence of each predictor in the final model. An "X" indicates the effect was included in the model, while a "-" indicates the effect was absent. ***/**/*: importance of variable as a predictor of admittance to centre.

	Total		
AICC	4471,066	4417,986	4418,457
Centre***	X	-	X
Year**	X	X	-
Temperature*	X	X	X
Null	-	-	-
	**Young bats**		
AICC	4415,218	4416,37	4416,708
Centre***	X	-	X
Year**	X	X	-
Temperature**	X	X	X
Null	-	-	-
	**Adults**		
AICC	-54,41	-52,599	-52,575
Centre**	X	X	X
Year*	X	X	X
Temperature	-	-	X
Null	-	X	-
